# The Relationship between Online Social Networking and Sexual Risk Behaviors among Men Who Have Sex with Men (MSM)

**DOI:** 10.1371/journal.pone.0062271

**Published:** 2013-05-01

**Authors:** Sean D. Young, Greg Szekeres, Thomas Coates

**Affiliations:** 1 Department of Family Medicine, David Geffen School of Medicine, University of California Los Angeles, Los Angeles, California, United States of America; 2 Program in Global Health, David Geffen School of Medicine, University of California Los Angeles, Los Angeles, California, United States of America; 3 Program in Global Health, David Geffen School of Medicine, University of California Los Angeles, Los Angeles, California, United States of America; Manchester University, United Kingdom

## Abstract

Online social networking usage is growing rapidly, especially among at-risk populations, such as men who have sex with men (MSM). However, little research has studied the relationship between online social networking usage and sexual risk behaviors among at-risk populations. One hundred and eighteen Facebook-registered MSM (60.1% Latino, 28% African American; 11.9% other) were recruited from online (social networking websites and banner advertisements) and offline (local clinics, restaurants and organizations) venues frequented by minority MSM. Inclusion criteria required participants to be men who were 18 years of age or older, had had sex with a man in the past 12 months, were living in Los Angeles, and had a Facebook account. Participants completed an online survey on their social media usage and sexual risk behaviors. Results from a multivariable regression suggest that number of sexual partners met from online social networking technologies is associated with increased: 1) likelihood of having exchanged sex for food, drugs, or a place to stay within the past 3 months; 2) number of new partners within the past 3 months; 3) number of male sex partners within the past 3 months; and 4) frequency of engaging in oral sex within the past 3 months, controlling for age, race, education, and total number of sexual partners. Understanding the relationship between social media sex-seeking and sexual risk behaviors among at-risk populations will help inform population-focused HIV prevention and treatment interventions.

## Introduction

Within the United States, a disproportionate number of incident HIV infections and new diagnoses of HIV (e.g., cases detected long after incident infection) lie within men who have sex with men (MSM). In Los Angeles, for instance, over 75% of all infections are attributable to MSM [1], [2], [3]. Among MSM in the United States, Latinos accounted for 20% of new infections and African Americans accounted for 37% of new infections [4]. To address the epidemic among at-risk populations, researchers have requested innovative approaches for understanding and preventing sexual risk behaviors.

Online technologies have been rapidly growing in use and may play a role in facilitating sexual risk behaviors, especially among at-risk populations [5], [6]. Internet use was previously used primarily among upper-middle-class white populations, however, minority groups have increasingly used online technologies [7]. For instance, in the United States, from 2005 to 2006, there was a 121% increase in the number of African American households with high-speed Internet connections and a 46% increase in Latino households with broadband usage, with 56% of Latinos using the Internet on a daily basis [8], [9]. In fact, English-speaking Latinos are almost identical to Whites in their use of Internet and home broadband [10], and African Americans and Latinos are more likely than Whites to access the Internet from mobile devices [11].

Social networking technologies, platforms for virtual social communication, have been the predominant factor for the growth in online technology use among minority groups. Social networking sites, such as Facebook, Grindr, and Twitter, are online platforms designed for social communication through sharing of pictures, messages, and other forms of multi-media communication [12]. African Americans and English-speaking Latinos are almost 1.5 times more likely to use online social networking sites compared to the general adult population (33% of African Americans, 36% of English-speaking Latinos, and 23% of adults in the general population). This trend is consistent in Twitter usage, online video usage, and usage of location-based devices [13]. In addition, gay, lesbian, and bisexual individuals use social networking technologies more often than heterosexual individuals [9].

Although research has explored the link between Internet use and sexual risk behaviors [14], [15], [16], [17], little work has focused on social networking use and sexual risk. However, it is especially important to study the relationship between social networking and sexual risk behaviors because these technologies were specifically designed for social (and potentially sexual) communication and interaction [12], [18]. Research from (non-social networking) Internet studies suggest that, compared to people who do not seek sex on the Internet, Internet sex seekers tend to have: more frequent anal sex, more previously diagnosed STIs, more sexual exposure to men, greater numbers of sex partners, and higher numbers of sex partners known to be HIV positive [14]. Social networking users have a potentially increased risk, as social networking technologies allow users to engage in real-time interaction that can be used to initiate rapid sexual encounters. For example, people can use location-based mobile applications such as SexMap (DoubleD, 2012) and Grindr (Nearby Buddy Finder, LLC, 2009) to search for potential sexual encounters with others in immediate geographic proximity. It is therefore important to evaluate how social networking technologies might affect sexual risk behaviors, especially among social networking users who are at high-risk for HIV, such as MSM.

This study seeks to determine the relationship between social networking use and sexual risk behaviors among minority MSM. Specifically, we assess 1) rates of using social networking technologies for sex-seeking, and 2) sexual risk behaviors associated with seeking sex on online social networks.

## Methods

This study was conducted according to the principles expressed in the Declaration of Helsinki. The UCLA Office of the Human Research Protection Program approved this study. Methods conform to recommended guidelines for using social networking technologies in HIV prevention research [19]. Participants provided a web-based informed consent.

Over a period of 4.5 months (September 2010-January 2011), 122 participants were recruited online, from physical venues frequented by African American and Latino MSM (e.g., bars, clubs, and universities), and from direct referrals from study participants. Participants were paid $30 in gift cards to complete a survey. Four participants were found to have completed multiple surveys. Their second response was dropped, leaving 118 participant responses. Six participants completed part of the survey. Their responses (when available) are included in this analysis.

Participants were recruited online using the following methods: (1) paid, targeted banner ads on social networking sites (Facebook.com, Myspace.com), and (2) setting up a fan page on Facebook with information describing the study. Participants recruited using online methods were directed to a website where they could receive more information and enroll.

Fliers placed in physical venues frequented by African American and Latino MSM described the study and provided a contact email address and a web link for participants to receive more information and enroll. Most fliers were culturally tailored, including a picture of a male Latino or African American, stating that the study was looking for male participants who were 18 years of age or older, African American or Latino, interested in men, and had a Facebook account. Fliers provided a contact email address and link to a Website where participants could receive more information and enroll. Participants were informed that they could refer friends who were interested and fit the inclusion criteria. Participants were not paid additional incentives to refer friends.

Potential participants visited the study Website and were screened for eligibility. Eligible participants were males who were: 18 years of age or older, living in Los Angeles, had had sex with a man in past 12 months, and had a Facebook account. Because we attempted to recruit a sample of predominantly minority MSM, we first recruited 70% of the sample from these populations and then opened recruitment to MSM from other populations. Participants were excluded if they did not fit inclusion criteria. A Facebook Connect application was used as a verification tool to ensure that each participant had a registered and unique Facebook account. If the eligible participant consented to the study, he had to provide his Facebook username and password through Facebook Connect. Once this connection method verified his status as a Facebook participant, he was asked to input his email address, phone number, and completed an online survey.

The 92-item online survey took approximately 45 minutes and included a collection of items from previous research on sexual risk behaviors, Internet use, as well as a number of novel items related to use of social networking technologies. Items focused on demographics (age, gender (to ensure that all participants reported being men), race/ethnicity, income, and education); Internet and social media usage; and sexual health behaviors (see Participant Questionnaire). Prior to responding to items related to social media use, participants were given a definition of social networking sites along with a list of examples sites (such as Facebook and Myspace). Internet and social media usage items focused on the amount of time spent using the Internet and social media (in hours per day, days per week); reasons for using these technologies (e.g., news, dating, finding sex partners); and comfort when using these technologies to talk about sexual risk behaviors. For example, after a description of the difference between general Internet sites and social networking sites, participants were asked about their use of social networking sites for seeking sex, “In the past 3 months, how many sexual partners have you met on the Internet/social networking sites?” They were not asked to differentiate between general social networking websites and websites that were designed specifically for dating or seeking sex. Sexual risk behavior-related items focused on number and gender of sexual partners (from online and offline sources), sexual behaviors, and number of times exchanging sex for food, drugs, or a place to stay. For example, participants were asked, “In the past 3 months, have you exchanged sex for food, drugs, or a place to stay?”

Chi-square tests were used to assess differences in population demographics, Internet and social media usage, and sexual risk behaviors. Analysis of variance (ANOVA) tests were used to assess differences in age between groups as well as to confirm Chi-square differences on continuous variables. Multiple regression analysis was used to assess the relationship between number of sex partners met on the social networks and exchanged sex, new sex partners, sex with men, and oral sex, controlling for age, race, education, and total number of sex partners. Total number of sex partners was added to control for the possibility that group differences in social networking sex partners could be accounted for by overall increases in sex partners. Analyses were performed on de-identified data using Stata software [20].

## Results

As Facebook Connect was used to verify Facebook status, 100% of participants were current Facebook users. Slightly less than half of the sample used Myspace (45.5%) and Twitter (44.6%), with a smaller portion using Grindr (18.8%) and various sex-seeking sites such as Adam4Adam.com and Manhunt.com (18.8%).

Table s1 displays the demographic results between populations and in the overall sample. Participants were predominantly Latino (60.1%) or African American (28%). The majority of participants had at least a high school education, with over 60% of participants from the western United States. Almost all participants reported being either gay (76.3%) or bisexual (17.8%), and single (82.2%). The average age of the sample was just under 32 years of age, with White participants on average being older than the rest of the sample. Group differences by population were found on education level, birthplace, and age.

The majority of participants had used the Internet and online social networking technologies to meet new sex partners within the past 3 months. On average, participants met over 4 of their most recent sex partners using these technologies. Compared to African Americans, Latinos on average more frequently used Internet/social networking technologies to meet sex partners (Tables s2 and s3).

Table s4 presents results of a regression analysis looking at exchanged sex, new sex partners, sex with men, and oral sex as outcomes. Controlling for age, race, education, and total number of sex partners, there was a significant positive relationship between number of sex partners met from online social networking technologies and 1) likelihood of having exchanged sex for food, drugs, or a place to stay, 2) number of new sex partners within the past 3 months, 3) number of male sex partners within the past 3 months, and 4) likelihood of engaging in oral sex.

## Discussion

Results from an analysis of MSM social networking users suggest that MSM are using online social networking technologies for sex-seeking, and that meeting sexual partners from social networking sites is associated with increased likelihood of engaging in sexual risk behaviors. Those who met sexual partners from social networking sites were more likely than those who did not to have exchanged sex for food, drugs, or a place to stay; have new sex partners within the past 3 months; have had sex with a greater number of men; and have engaged in oral sex. These effects were not due to the possibility that people who engage in more frequent sexual encounters engage in more sexual risk behaviors (as we controlled for overall number of sex partners), but were unique to sex-seeking on social networking sites. These results are important because they suggests that social networking websites, when used for sex-seeking, may be associated with transmission of HIV and other sexually transmitted infections.

The ability to recruit African American and Latino MSM Facebook users for this study provides support that minority groups are actively and increasingly using social media technologies.[10], [13] This study builds on those findings by suggesting that minority MSM are actively using social networking technologies for seeking sex. Knowledge of these changing trends is useful for understanding both the (virtual and physical) locations and behaviors of at-risk groups so that these same technologies that could potentially facilitate HIV transmission could also be used for HIV prevention. Latino MSM appear to be especially likely to use social networking technologies to search for sex partners. This finding could be useful in helping Latino MSM researchers understand how to use social networking technologies for Latino MSM recruitment and for development of culturally-tailored HIV prevention interventions.

This study builds on research on the relationship between Internet use and HIV risk [14], [16], [21], [22] by showing that number of sex partners met on social networking sites was associated with increased likelihood of engaging in sexual risk behaviors (e.g., exchanging sex and meeting new sex partners). However, number of sex partners from social networking sites was also associated with an increased likelihood of having had oral sex. This finding may provide support that the social networking sites do not necessarily lead to increased HIV transmission, as people using the technologies for sex-seeking might be able to meet and have sex with more individuals but might engage in oral sex to mitigate these risks [23].

This study provides new data on the use of social networking technologies among at-risk groups. It is important to begin to understand how use of social networking technologies, by way of promoting both social engagement and anonymity, can affect HIV risk behaviors. Studying how social networking users engage other sex partners will be important in crafting HIV prevention interventions toward at-risk populations. For example, websites that track and integrate geolocation technologies (such as Grindr) are currently being used to initiate rapid sex-seeking encounters. As technology develops and can be used to facilitate sex-seeking and sexual risk behaviors, it becomes important that researchers and policymakers study and understand how these same technologies can be used to prevent the spread of HIV and other sexually transmitted infections. Policymakers and sexual health organizations have already begun using social media to reach at-risk populations, and as research is furthered in this area it can be used to support models for improvements in sexual health policies and interventions.

Study limitations are based primarily on the focused population and enrollment criteria. First, as the sample is based in Los Angeles, it is possible that participants from other locations might not share the same population characteristics. However, high HIV rates in Los Angeles make Los Angeles an important area for HIV prevention research. Second, although sexual risk behaviors presented in this study are associated with HIV transmission, knowing the specific type of sexual behavior would help to more accurately determine the associated risk. For example, while exchanging sex for food and drugs is associated with sexual risk, engaging in unprotected anal intercourse in exchange for drugs would be associated with greater risk than engaging in protected intercourse. This study presents a first look at associations between social networking use and sexual risk behavior, and future studies that identify the specific sexual behaviors (such as protected versus unprotected sexual intercourse) may help to provide a more definitive link to HIV transmission. Next, participants were not asked to report how they were recruited to the study. Future research can address this question to provide data on best recruitment methods. Finally, it is possible that the present results might not generalize to groups other than MSM, or early technology adopters. Although social networking use is being studied among broader racial and ethnic populations of people living in the Untied States[10], [13], research on the link between social networking and HIV risk has only recently been studied and has focused on populations at high-risk for HIV, such as MSM [19], [24]. Future research is encouraged to test whether these findings extend to other at-risk populations.

## Conclusion

The present study suggests that African American and Latino MSM are using social networking technologies to search for and meet sex partners, and those who use these technologies are more likely to engage in HIV risk behaviors. Understanding how at-risk populations use social networking technologies is important for crafting and scaling culturally-tailored HIV prevention interventions.

## Supporting Information

Table S1 **Socio-demographic Characteristics of Study Participants (N = 118), Los Angeles, CA, 2011.**
(DOC)

Table S2 **Internet and sex-related behaviors (N  =  118), Los Angeles, CA 2011.**
(DOC)

Table S3 **Online sex seeking and sexual behaviors, by race (N  = 118), Los Angeles, CA, 2011.**
(DOC)

Table S4 **Analysis of exchanged sex, new sex partners, sex with men, and oral sex, (N  = 118), Los Angeles, CA, 2011.**
(XML)
