# Supplementary material for: The Relationship between Online Social Networking and Sexual Risk Behaviors among Men Who Have Sex with Men (MSM)
Source: PLoS One. 2013 May 1;8(5):e62271. doi: 10.1371/journal.pone.0062271 (PMC3642936; doi:10.1371/journal.pone.0062271)
Supplement: Table S1 — Socio-demographic Characteristics of Study Participants (N = 118), Los Angeles, CA, 2011. (DOC) [file pone.0062271.s001.doc]

**Table s1. Socio-demographic Characteristics of Study Participants (N=118), Los Angeles, CA, 2011**

|  |  | African American (n = 33) | Latino (n = 71) | Other (n= 14) | | Chi Square | Total Sample |
| --- | --- | --- | --- | --- | --- | --- | --- |
| Participant population (%) |  | 28.0 | 60.2 | 11.9 | |  | 100 |
| Highest Education* | | | | | | | |
|  | Less than HS | 3 | 4.2 | | 0 |  | 3.4 |
|  | HS | 36.4 | 31 | | 16.7 |  | 30.5 |
|  | GED | 0 | 7 | | 0 |  | 5.1 |
|  | Associates | 12.1 | 28.2 | | 16.7 |  | 22 |
|  | Bachelors | 24.2 | 23.9 | | 50.1 |  | 27.1 |
|  | Graduate School | 24.2 | 5.6 | | 16.7 | 26.5 | 11.9 |
| Birthplace** |  |  |  | |  |  |  |
|  | Northern USA | 21.2 | 15.5 | | 0 |  | 15.3 |
|  | Southern USA | 15.2 | 8.5 | | 8.3 |  | 10.2 |
|  | Eastern USA | 15.2 | 0 | | 16.7 |  | 5.9 |
|  | Western USA | 48.4 | 69 | | 66.9 |  | 62.7 |
|  | Latin America/ Caribbean | 0 | 7 | | 8.3 | 77.71 | 5.1 |
| Self-Described Sexual Orientation |  |  |  | |  |  |  |
|  | Homosexual | 54.55 | 87.32 | | 75.1 |  | 76.3 |
|  | Bisexual | 39.4 | 7 | | 16.8 |  | 17.8 |
|  | Questioning | 6.1 | 5.6 | | 8.2 | 22.24 | 5.9 |
| Current Marital Status |  |  |  | |  |  |  |
|  | Single | 84.9 | 81.7 | | 75.2 |  | 82.2 |
|  | Married/ Partnered | 6 | 14.1 | | 16.6 |  | 11.8 |
|  | Divorced/ Other | 9.1 | 4.2 | | 8.2 | 6.22 | 6.0 |
|  |  |  |  | |  | F-statistic | |
| Age (mean, SD)** | | 33.8, 9.6 | 29.5, 6.4 | | 40.7, 10.5 | 8.2 | 31.8 |

* Significant at the *p* <.05 level

** Significant at the *p* < .01 level
