# Supplementary material for: The Relationship between Online Social Networking and Sexual Risk Behaviors among Men Who Have Sex with Men (MSM)
Source: PLoS One. 2013 May 1;8(5):e62271. doi: 10.1371/journal.pone.0062271 (PMC3642936; doi:10.1371/journal.pone.0062271)
Supplement: Table S2 — Internet and sex-related behaviors (N = 118), Los Angeles, CA 2011. (DOC) [file pone.0062271.s002.doc]

| **Table s2. Internet and sex-related behaviors among Facebook users (N = 118), Los Angeles, CA 2011** | | | | | | |
| --- | --- | --- | --- | --- | --- | --- |
| In the past 3 months, have you used online social networks to meet new sexual partners? | | | | | | |
|  |  | | N | % |  | |
|  | No | | 49 | 43.75 | |  |
|  | Yes | | 63 | 56.25 | |  |
| In the past 3 months, how many of your sexual partners were people you met on the Internet/social networking sites? | | | | | | |
|  | Mean (SE) | | 4.1 | 0.03 |  | |
|  | | | | |  | |
| In the past 3 months, how many different men have you had sex with? | | | | | | |
|  | | Mean (SE) | 5.8 | 1.15 |  | |
|  | | | | | | |
| In the past 3 months, how many of your sexual partners have been new sexual partners (total number of new sex partners in past 3 months)? | | | | | | |
|  | | Mean (SE) | 4.1 | 1.07 |  | |
