# Supplementary material for: The Relationship between Online Social Networking and Sexual Risk Behaviors among Men Who Have Sex with Men (MSM)
Source: PLoS One. 2013 May 1;8(5):e62271. doi: 10.1371/journal.pone.0062271 (PMC3642936; doi:10.1371/journal.pone.0062271)
Supplement: Table S3 — Online sex seeking and sexual behaviors, by race (N = 118), Los Angeles, CA, 2011. (DOC) [file pone.0062271.s003.doc]

Table s3. Online sex seeking, sexual behaviors, and honesty talking online to others about sex behaviors, by race (N =118), Los Angeles, CA, 2011

|  | African American | | Latino | | Other | |
| --- | --- | --- | --- | --- | --- | --- |
|  | Mean | Standard Error | Mean | Standard Error | Mean | Standard Error |
| Used social networks to find sex partners (n, %)* | 14 | 46.7% | 41 | 60.3% | 8 | 57.1% |
| Number of sex partners met on online social networks | 3.4 | 0.8 | 4.6 | 1.5 | 3.3 | 1.5 |
| Total number of male sex partners | 5.6 | 1.8 | 6.4 | 1.7 | 3.3 | 0.89 |
| Total number of new sex partners | 3 | 0.7 | 4.9 | 1.8 | 2.5 | 0.72 |

*This variable is expressed in number of participants and sample percentage. Percentages refer to percentage of participants within each racial group
